# A Longitudinal Study of Older Adults’ Acceptance of Robot Companions and Their Effects on Well-Being

**DOI:** 10.1093/geroni/igab046.152

**Published:** 2021-12-17

**Authors:** Claudia Rebola, Bertram Malle

**Affiliations:** 1 University of Cincinnati, Cincinnati, Ohio, United States; 2 Brown University, Brown University, Rhode Island, United States

## Abstract

Robotic animal-like companions for older adults are promising technologies that have shown to have health benefits, especially for individuals with dementia, and good adoption rates in some previous studies. Our project, Affordable Robotic Intelligence for Elderly Support, aims to design new capabilities for companionship and smart care, but at high affordability. In a 6-month longitudinal study of baseline acceptance and well-being, we assessed the impact of an Ageless Innovation Joy for All™ robotic pet on user acceptance and emotional well-being (depression, loneliness, positive emotions). Nineteen participants from independent and assisted living facilities completed three standardized in-person surveys, each 3 months apart, including the CES-D, measures of Loneliness, Emotions, Attitude towards Technology (ATI), and various measures of evaluation of and engagement with robotic technology. The measures showed modest to very good reliability and meaningful construct validity. Participants in this sample showed little depression or loneliness, and these levels did not further decrease over the six months. People welcomed the pet and expressed positive evaluations of it, and these sentiments were stable over time. Attitudes toward technology varied but were unrelated to well-being measures and to robot evaluations. Our current conclusion, on the basis of a small sample, is that the selected robotic pet companion is appreciated and seen as beneficial, and for adults who are already low in depression and loneliness, the robot companion helps maintain the adult’s emotional well-being but does not further increase it.

